# Progress in prevention of mother-to-child transmission of HIV infection in Ukraine: results from a birth cohort study

**DOI:** 10.1186/1471-2334-9-40

**Published:** 2009-04-07

**Authors:** Claire Thorne, Igor Semenenko, Tatyana Pilipenko, Ruslan Malyuta, the Ukraine European Collaborative Study Group

**Affiliations:** 1 MRC Centre of Epidemiology for Child Health, UCL Institute of Child Health, University College London, UK; 2 Perinatal Prevention of AIDS Initiative, Odessa, Ukraine

## Abstract

**Background:**

Ukraine was the epicentre of the HIV epidemic in Eastern Europe, which has the most rapidly accelerating HIV epidemic world-wide today; national HIV prevalence is currently estimated at 1.6%. Our objective was to evaluate the uptake and effectiveness of interventions for prevention of mother-to-child transmission (PMTCT) over an eight year period within operational settings in Ukraine, within the context of an ongoing birth cohort study.

**Methods:**

The European Collaborative Study (ECS) is an ongoing birth cohort study in which HIV-infected pregnant women identified before or during pregnancy or at delivery were enrolled and their infants prospectively followed. Three centres in Ukraine started enrolling in 2000, with a further three joining in September 2006.

**Results:**

Of the 3356 women enrolled, 21% (689) reported current or past injecting drug use (IDU). Most women were diagnosed antenatally and of those, the proportion diagnosed in the first/second trimester increased from 47% in 2000/01 (83/178) to 73% (776/1060) in 2006/07 (p < 0.001); intrapartum diagnosis was associated with IDU (Adjusted odds ratio 4.38; 95%CI 3.19–6.02). The percentage of women not receiving any antiretroviral prophylaxis declined from 18% (36/205) in 2001 to 7% in 2007 (61/843) (p < 0.001). Use of sdNVP alone substantially declined after 2003, with a concomitant increase in zidovudine prophylaxis. Median antenatal zidovudine prophylaxis duration increased from 24 to 72 days between 2000 and 2007. Elective caesarean section (CS) rates were relatively stable over time and 34% overall. Mother-to-child transmission (MTCT) rates decreased from 15.2% in 2001 (95%CI 10.2–21.4) to 7.0% in 2006 (95%CI 2.6–14.6). In adjusted analysis, MTCT risk was reduced by 43% with elective CS versus vaginal delivery and by 75% with zidovudine versus no prophylaxis.

**Conclusion:**

There have been substantial improvements in use of PMTCT interventions in Ukraine, including earlier diagnosis of HIV-infected pregnant women and increasing coverage with antiretroviral prophylaxis and the initial MTCT rate has more than halved. Future research should focus on hard-to-reach populations such as IDU and on missed opportunities for further reducing the MTCT rate.

## Background

Ukraine has the most severe HIV epidemic in the whole of Europe and the Commonwealth of Independent States with an estimated 440,000 people living with HIV/AIDS and an adult HIV prevalence rate of 1.6% [1-3]. Ukraine was the epicentre of the HIV epidemic in the region [4] and although injecting drug use (IDU) was the main driver of the epidemic, heterosexual transmission has subsequently increased substantially [5-8]. The increased heterosexual transmission of HIV is closely linked to risky sexual behaviour among and with injecting drug users [3]. Women account for nearly half of the infected population, with an estimated 190,000 women living with HIV/AIDS, the vast majority in their child-bearing years [8].

Most HIV infections in children occur through mother-to-child transmission (MTCT), which can take place in utero, intrapartum and through breastfeeding [9]. The WHO/UNAIDS Strategic response to prevention of HIV infection in infants involves four "pillars"-primary prevention of infection in women, prevention of unintended pregnancies in HIV-infected women, prevention of transmission from HIV-infected women to their infants and provision of treatment, care and support to HIV-infected women and their families [10,11]. Interventions effective for the prevention of MTCT (PMTCT) include antiretroviral (ARV) prophylaxis, elective caesarean section (CS) and avoidance of breastfeeding [12]. In resource-rich settings, where antenatal highly active antiretroviral therapy (HAART) is extensively used, MTCT rates have declined from around 16–25% in non-breastfeeding women to less than 1–2% [13-15]. In resource-limited settings, various abbreviated ARV prophylactic regimens have been shown to be effective PMTCT interventions, with single dose nevirapine (sdNVP) the most commonly used to date, although concerns exist regarding emergence of resistance following sdNVP exposure [12,16].

Ukraine adopted its first Programme for Preventing HIV in Newborns in 2001 [17], involving an opt-out universal antenatal HIV testing policy, sdNVP for infected mothers and their infants and provision of free infant formula. Following a review in 2003, the programme was updated with zidovudine (ZDV) starting from at least 28 weeks gestation and one week of neonatal ZDV prophylaxis to replace sdNVP; for women identified as HIV-infected after 28 weeks gestation, the addition of sdNVP to ZDV for mother and infant was recommended, while infants of mothers identified intrapartum should also receive extended (four weeks) ZDV prophylaxis [18].

Our aim was to evaluate the uptake and effectiveness of PMTCT interventions over an eight year period in Ukraine, within a multicentre cohort study of HIV-infected pregnant women and their infants, the European Collaborative Study (ECS).

## Methods

### Study setting and subjects

The ECS is an ongoing birth cohort study, in which HIV-1 infected pregnant women are enrolled and their infants prospectively followed according to a standard protocol. The ECS was established in 1985 in Western Europe to estimate the rate of and risk factors for MTCT [19]. Centres from Ukraine joined in 2000: sites in Odessa, Mykolaiv and Simferopol started enrolling at the start of 2000 and those in Kiev, Donetsk and Mariupol in September 2006 [15].

In Ukraine, antenatal care services are free of charge (including HIV testing), and antenatal care coverage increased from 90% in 1999 to 99% in 2007, with almost all deliveries taking place in health facilities. For 2003–2007, the infant mortality estimate was 14 per 1,000 live births [20]. Pregnant women in Ukraine are screened for HIV infection at pregnancy registration and for those testing negative there is repeat testing at around 30 weeks gestation. HIV testing is free and included within the standard antenatal care package. A national policy for offering rapid testing during labour for women who have presented with unknown HIV status, was introduced in 2003, those who were tested positive received sdNVP prophylaxis. All pregnant women identified as HIV-infected before or during pregnancy, or through intrapartum testing, were invited to participate in the ECS, with informed consent. Data are collected anonymously on standard questionnaires, using study serial numbers, without personal identifiers (linked anonymous data). Information collected includes maternal socio-demographic and clinical information, delivery and infant characteristics. Flow cytometry became available in Odessa and Simferopol in 2004; Donetsk and Kiev had this capacity from their first enrolments in autumn 2006. HIV-infected infants were diagnosed based on persistence of HIV antibody beyond age 18 months up until the start of 2006; subsequently, early diagnosis of HIV-exposed infants with PCR testing was introduced nationwide, with facilities in three inter-regional laboratories. However, coverage (both nationally and within ECS sites) remains patchy due to slow uptake of the rapid testing service in some AIDS centres and a period when no rapid testing was available at one of the inter-regional laboratories due to technical problems [21]; 40% (276/698) of infants born in 2006 and enrolled in the ECS had PCR test results available.

### Definitions

Infants with persistence of antibody beyond 18 months of age and/or a positive virological marker of infection regardless of age were included as HIV-infected. If a child was HIV antibody-negative and no virus had been detected, (s)he was classified as uninfected, regardless of age. Elective CS deliveries were classified as occurring before rupture of membranes and onset of labour. Premature delivery was defined as occurring before 37 completed weeks of gestation. Injecting drug use (IDU) history (current or past) was assigned according to self-report, clinical report or neonatal drug withdrawal symptoms. Maternal moderate/severe HIV symptoms were defined as those in WHO Clinical stage 3 or 4. Multiple births (32 twin pairs) were treated as separate mother-child pairs.

### Data analysis

Univariable comparisons were assessed with the χ^2 ^test for categorical variables. Logistic regression was used to obtain unadjusted odd ratios (OR) and adjusted odds ratios (AOR) and 95% confidence intervals (CI) in analyses identifying factors associated with timing of maternal HIV diagnosis and to investigate risk factors for non-receipt of ARV prophylaxis and for MTCT. The analyses for factors associated with pre-pregnancy diagnosis were limited to 2871 women with complete data available on: mode of HIV acquisition, time period, maternal age and HIV symptoms. The regression analyses of intrapartum maternal diagnosis included the above variables and were limited to 2336 women with unknown HIV status at conception. The analysis investigating non-receipt of ARV prophylaxis included time of maternal HIV diagnosis, maternal age, mode of acquisition, gestational age and time period, with 3224 women with complete data included. The analysis of MTCT risk included 1635 mother-child pairs with known infant infection status and no missing information on the explanatory variables (prematurity, mode of delivery, ARV prophylaxis, IDU history and breastfeeding). Statistical analyses were performed with SAS (v8.02, SAS Institute, Cary, North Carolina, USA).

#### Ethics approval

The ECS has been approved by the Great Ormond Street Hospital for Children NHS Trust/Institute of Child Health Ethics Committee.

## Results

### HIV-infected pregnant women

By January 2008, 3356 mother-child pairs had been enrolled. Most women were young, nulliparous, married or cohabiting and reported no specific HIV acquisition risk factors (Table 1). Fifteen (0.5%) women were born outside Ukraine, mostly elsewhere in the Commonwealth of Independent States. Of the 689 women with an IDU history, 357 (52%) were current and 332 (48%) previous users. The proportion of women reporting an IDU history declined from 36% (88/245) in 2000/01 to 29% (152/530) in 2002/03, 24% (230/978) in 2004/05 and 14% (219/1537) in 2006/07 (χ^2^_trend _= 96.1, p < 0.001).

**Table 1:** Characteristics of the HIV-infected pregnant women and their infants

	N (%)
**Median age **(range)	25.6 years (14–44)
**Parity at enrolment **(n = 3324)	
0	2026 (61)
1	966 (29)
≥2	332 (10)
**History of pregnancy termination **(n = 3325)	
0	1948 (59)
1	715 (21)
≥2	662 (20)
**Age at leaving full-time education**	
Median (IQR)	17 years (16–19)
**Marital status **(n = 3328)	
Married	1372 (41)
Cohabiting	1324 (40)
Single, divorced, widowed	632 (19)
**Mode of acquisition of HIV **(n = 3356)	
Injecting drug use	689 (21)
Injecting drug using partner	735 (22)
Other heterosexual contact	375 (11)
Other risk	9
No specific risk factor reported	1548 (47)
**Timing of mother's first positive HIV test **(n = 3356)	
Before pregnancy	893 (27)
First/second trimester	1517 (45)
Third trimester	631 (19)
In pregnancy (date not specified)	81 (2)
Delivery	234 (7)
**Maternal HIV clinical stage **(n = 2956)	
Asymptomatic/mild symptoms	2838 (96)
Moderate/severe symptoms	118 (4)
**Gestational age **(n = 3354)	
<37 weeks	298 (9)
≥37 weeks	3056 (91)
**Birth weight **(n = 3354)	
Median (range)	3100 g (850–5000 g)
**Neonatal prophylaxis (n = 3356)**	
None	484 (14)
sdNVP only	773 (23)
ZDV	1907 (57)
sdNVP and ZDV	121 (4)
Type not recorded	71 (2)

### Timing of maternal HIV diagnosis

Most women received their first HIV diagnosis in pregnancy (Table 1). The proportion of women with a pre-pregnancy HIV diagnosis increased slightly from 25% (58/236) in 2000/01 to 31% (476/1536) in 2006/07, whilst the proportions diagnosed in the third trimester and intrapartum more than halved, decreasing in the same years from 30% (71/236) to 14% (219/1536) and from 10% (24/236) to 4% (65/1536) respectively. Among women with unknown HIV status at the start of pregnancy, the proportion diagnosed in the first or second trimester increased from 47% in 2000/01 (83/178) to 73% (776/1060) in 2006/07 χ^2 ^= 49.4, p < 0.001).

Factors associated with mothers having received a pre-pregnancy HIV diagnosis were investigated (Table 2). An IDU history, having an IDU sexual partner, moderate/severe HIV symptoms and older age were significantly associated with pre-pregnancy diagnosis; additionally, women enrolling in 2006/07 were nearly twice as likely to know their infection status before pregnancy as those enrolling in 2000/01. Maternal HIV diagnosis intrapartum was associated with IDU, with IDUs having a four-fold increased risk of diagnosis at this late stage than other women (Table 3). The reduced likelihood of intrapartum diagnosis in the later years of the study lost statistical significance in the adjusted model.

**Table 2:** Factors associated with diagnosis of HIV infection prior to pregnancy

	N	N (%) diagnosed pre-pregnancy	Odds ratio (95% CI)	Adjusted odds ratio (95% CI), *p *value
**Mode of acquisition**				
Non IDU, no IDU sex partner	1573	338 (21)	1.00	1.00
Non IDU, IDU sex partner	696	195 (28)	1.42 (1.16–1.75)	1.46 (1.19–1.80) *p *< 0.001
IDU	601	263 (44)	2.85 (2.33–3.48)	2.94 (2.38–3.63) *p *< 0.001
				
**Maternal age**				
<25 years	1303	298 (23)	0.65 (0.52–0.80)	0.79 (0.63–0.99) *p *= 0.04
25–30 years	939	300 (32)	1.02 (0.82–1.27)	1.12 (0.90–1.41) *p *= 0.31
≥30 years	629	198 (31)	1.00	1.00
				
**Maternal moderate/severe HIV symptoms**				
No	2753	733 (27)	1.00	1.00
Yes	118	63 (53)	3.16 (2.18–4.58)	2.32 (1.57–3.42) *p *< 0.001
				
**Time period of delivery**				
2000–2001	187	45 (24)	1.00	1.00
2002–2003	431	99 (23)	0.94 (0.63–1.41)	1.06 (0.70–1.60) *p *= 0.8
2004–2005	977	243 (25)	1.05 (0.73–1.51)	1.19 (0.81–1.74) *p *= 0.37
2006–2007	1276	409 (32)	1.49 (1.04–2.12)	1.87 (1.29–2.72) *p *< 0.001

95%CI – 95% confidence interval

**Table 3:** Factors associated with intrapartum diagnosis of HIV infection

	N	N (%) diagnosed intra-partum	Odds ratio (95% CI)	Adjusted odds ratio (95% CI), *p *value
**Mode of acquisition**				
Non IDU, no IDU sex partner	1433	97 (7)	1.00	1.00
Non IDU, IDU sex partner	523	26 (5)	0.72 (0.46–1.12)	0.73 (0.47–1.14) *p *= 0.17
IDU	380	99 (26)	4.85 (3.57–6.60)	3.62 (2.61–5.03) *p *< 0.001
				
**Maternal age**				
<25 years	1117	75 (7)	1.00	1.00
25–30 years	727	73 (10)	1.55 (1.11–2.17)	1.30 (0.92–1.85) *p *= 0.17
≥30 years	492	74 (15)	2.46 (1.75–3.46)	1.90 (1.32–2.72) *p *< 0.001
				
**Marital status**				
Single, divorced, widowed	425	69 (16)	1.00	1.00
Married or cohabiting	1911	153 (8)	0.45 (0.33–0.61)	0.55 (0.39–0.76) *p *< 0.001
				
**Time period of delivery**				
2000–2001	169	21 (12)	1.00	1.00
2002–2003	373	49 (13)	1.07 (0.62–1.84)	1.27 (0.72–2.26) *p *= 0.40
2004–2005	734	87 (12)	0.95 (0.57–1.58)	1.16 (0.67–1.98) *p *= 0.60
2006–2007	1060	65 (6)	0.46 (0.27–0.78)	0.66 (0.38–1.15) *p *= 0.14

95%CI – 95% confidence interval

### Use of antenatal and intrapartum antiretroviral prophylaxis

**Figure 1:**
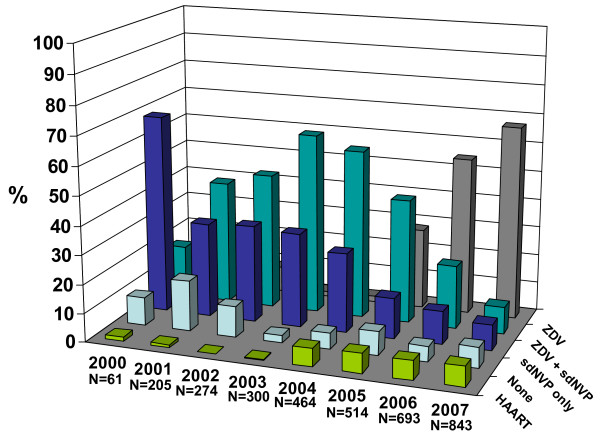
**Trends in use of antenatal and intrapartum antiretroviral prophylaxis/treatment**.

Overall, 93% of women received ARV drugs in pregnancy or in labour (Table 1) and Figure 1 illustrates the time trends in use of prophylaxis. The percentage of women receiving no prophylaxis declined from 18% (36/205) in 2001 to 7% in 2007 (61/843) (χ^2 ^= 11.0 p < 0.001). Intrapartum HIV diagnosis, premature delivery and IDU were associated with increased likelihood of women receiving no prophylaxis, whilst increasing calendar period was associated with a substantially decreased risk (Table 4). Women receiving both ZDV and sdNVP were more likely to have been diagnosed in the third trimester than women receiving ZDV alone (23% [245/1088] vs 11% [125/1126] χ^2 ^= 51.8, p < 0.001), reflecting national policy; duration of ZDV prophylaxis among women receiving ZDV+sdNVP was half of that among women receiving ZDV only (35 days vs 71 days). Median ZDV prophylaxis duration increased from 24 to 72 days between 2000 and 2007. The proportion of women taking antenatal ZDV for less than 31 days decreased from 62% in 2000/01 (63/101) and 2002/03 (204/330) to 24% (153/643) in 2004/05 and 7.5% (87/1160) in 2006/07 (χ^2^_trend _= 493.0, p < 0.001). Antenatal HAART use increased from 1.6% (1/61) in 2001 to 7% (60/844) in 2007. Of the 174 women on HAART, 16 (9%) had initiated this before pregnancy, with a median duration of 82 weeks (range 44–160) before pregnancy. The remaining women received HAART for a median of 11 weeks (range 1–36) during pregnancy.

**Table 4:** Factors associated with non-receipt of antenatal or intrapartum antiretroviral prophylaxis

	N	N (%) with non-receipt	Odds ratio (95% CI)	Adjusted odds ratio (95% CI) *p *value
**Time of maternal HIV diagnosis**				
Pre-pregnancy	886	93 (11)	1.00	1.00
1^st^/2^nd ^trimester	1500	18 (1)	0.10 (0.06–0.17)	0.12 (0.07–0.20) *p *< 0.001
3^rd ^trimester	617	50 (8)	0.75 (0.52–1.08)	0.79 (0.54–1.15) *p *= 0.21
Intrapartum	221	68 (31)	3.79 (2.65–5.42)	3.43 (2.34–5.01) *p *< 0.001
				
**Maternal age**				
<25 years	709	69 (10)	1.00	1.00
25–30 years	1453	84 (6)	0.57 (0.41–0.79)	0.87 (0.60–1.25) *p *= 0.45
≥30 years	1062	76 (7)	0.72 (0.51–1.01)	0.86 (0.59–1.24) *p *= 0.41
				
**Mode of acquisition**				
Non IDU, no IDU sex partner	1829	106 (6)	1.00	1.00
Non IDU, IDU sex partner	728	27 (4)	0.63 (0.41–0.96)	0.60 (0.38–0.93) *p *< 0.001
IDU	667	96 (14)	2.73 (2.04–3.66)	1.44 (1.03–2.01) *p *< 0.001
				
**Gestational age at delivery**				
≥37	2937	176 (6)	1.00	1.00
<37	287	53 (18)	3.55 (2.54–4.97)	2.21 (1.53–3.18) *p *< 0.001
				
**Time of delivery**				
2000–2001	224	38 (17)	1.00	1.00
2002–2003	488	26 (5)	0.28 (0.16–0.47)	0.23 (0.13–0.41) *p *< 0.001
2004–2005	978	68 (7)	0.37 (0.24–0.56)	0.37 (0.23–0.60) *p *< 0.001
2006–2007	1534	97 (6)	0.33 (0.22–0.50)	0.43 (0.28–0.68) *p *< 0.001

95%CI – 95% confidence interval

### Infants

Overall, 34% (n = 1153) of infants were delivered by elective CS, 3% (n = 104) by emergency CS and 63% (n = 2099) vaginally. Among mother-child pairs from the three centres enrolling since 2000 (n = 2809) elective CS rates increased from 30% (62/205) in 2001 to 41% (210/509) in 2007 (χ^2^_trend _= 12.6, p < 0.001). Overall, there were substantial centre differences, with an elective CS rate of 60% (410/689) in Simferopol versus 10% (11/115) in Kiev. Most neonates received ZDV prophylaxis (Table 1), of whom, 85% (1708/2016) received one week of ZDV syrup and 15% (n = 294) four weeks (no data on duration for 12 children). Type of antenatal and/or intrapartum prophylaxis was significantly associated with neonatal ZDV use, with neonatal prophylaxis taken by 44% (108/246), 35% (228/654), 68% (1546/2281) and 83% (145/174) of infants born to mothers who received no ARV prophylaxis, sdNVP only, antenatal ZDV and HAART respectively (χ^2 ^= 296.4, p < 0.001).

A total of 35 (1%) infants were breastfed; there was no significant trend over time, with 1.5% (4/267) breastfed in 2000/01, 0.7% (4/574) in 2002/03, 1.3% (13/978) in 2004/05 and 0.9% (14/1537) in 2006/07 (p = 0.6). Three of the breastfeeding mothers were diagnosed as HIV-infected following intrapartum rapid testing, 14 through antenatal testing and the remaining 18 (51%) diagnosed pre-pregnancy. Median breastfeeding duration among the 10 mother-child pairs with information available was 2 weeks (range 1–23).

### MTCT rates and risk factors

The overall MTCT rate was 11.4% (95%CI 9.9–13.0), with 192 children infected and 1498 uninfected; 1666 had indeterminate status, of whom 1132 (68%) were aged <18 months at the time of analysis. MTCT rates decreased from 15.2% in 2001 (26/171, 95%CI 10.2–21.4) to 7.0% in 2006 (6/86, 95%CI 2.6–14.6; limited to children aged ≥18 months old at last follow-up visit). Unadjusted MTCT rates by mode of delivery were 14.0% (141/1010, 95%CI 11.9–16.3) for vaginal deliveries, 19.3% (11/57, 95%CI 10.0–31.9) for emergency CS and 6.5% (40/623, 95%CI 4.7–8.8) for elective CS deliveries. Among women receiving no ARV prophylaxis, the unadjusted MTCT rate was 26.7% (32/120, 95%CI 19.0–35.5), decreasing to 15.7% (62/395, 95%CI 12.3–19.7) with sdNVP only, 7.0% (20/286, 95%CI 4.3–10.6) with ZDV, 9.2% (75/813, 95%CI 7.3–11.4) with ZDV and sdNVP and 3.9% (3/76, 95%CI 0.8–11.1) with HAART. Overall, IDUs had a significantly higher unadjusted MTCT rate compared with women with no IDU history (16.4% [67/409] vs 9.9% [121/1226] χ^2 ^= 12.2, p < 0.001), but within the IDU group MTCT rates were similar between women with current and prior use (16.5% [33/200] vs 16.3% [34/209] respectively, p = 0.95). Sixteen of the 35 breastfed infants had known infection status at last follow up, of whom 6 (37.5%) were infected (95%CI 15.2–64.6%).

Maternal HIV clinical disease stage (classified as asymptomatic/mild [WHO clinical stages 1 and 2] vs moderate/severe [stages 3 and 4]) was not significantly associated with MTCT risk (p = 0.96). MTCT risk more than doubled with premature delivery, increased 1.8-fold with maternal IDU history and five-fold with any breastfeeding in univariable analysis, while use of ARV prophylaxis and elective CS significantly decreased transmission risk (Table 5). In adjusted analysis, risk of transmission was reduced by 43% for women delivering by elective CS versus vaginal delivery and use of sdNVP halved transmission risk, with the reduction increasing to 86% for women on antenatal HAART (Table 5). Women receiving ZDV only were 72% less likely to transmit than those untreated (Table 5), and 50% less likely than women on sdNVP only (AOR 0.50; 95%CI 0.29–0.88, p = 0.02).

**Table 5:** Risk factors associated with mother-to-child transmission

	N	Odds ratio (95%CI)	Adjusted odds ratio (95%CI), *p *value
**Premature delivery**			
No	1491	1.00	1.00
Yes	144	2.22 (1.44–3.43) *p *< 0.001	1.43 (0.90–2.28) *p *= 0.13
**Mode of delivery**			
Vaginal	964	1.00	1.00
Emergency CS	57	1.44 (0.73–2.86) *p *= 0.29	1.58 (0.78–3.19) *p *= 0.20
Elective CS	614	0.42 (0.29–0.61) *p *< 0.001	0.57 (0.38–0.86) *p *= 0.007
**Antenatal/intrapartum ARVs**			
None	110	1.00	1.00
SdNVP	370	0.49 (0.30–0.81) *p *= 0.006	0.55 (0.33–0.92) *p *= 0.02
ZDV	280	0.20 (0.11–0.36) * p*< 0.001	0.28 (0.14–0.53) *p *< 0.001
ZDV with sdNVP	799	0.26 (0.16–0.42) *p *< 0.001	0.41 (0.24–0.69) *p *< 0.001
HAART	76	0.11 (0.03–0.36) *p *< 0.001	0.16 (0.05–0.54) *p *= 0.004
**IDU history**			
No	1266	1.00	1.00
Yes	409	1.79 (1.30–2.47) *p *< 0.001	1.31 (0.92–1.86) *p *= 0.13
**Breastfeeding**			
None	1620	1.00	1.00
Any	15	5.27 (1.85–15.0) *p *= 0.002	3.07 (1.02–9.24) *p *= 0.046

95%CI – 95% confidence interval

## Discussion

In this cohort of HIV-infected pregnant women and their infants, we have explored the effectiveness of the Ukraine PMTCT programme in operational settings. We have documented earlier diagnosis of HIV-infected pregnant women, increasing coverage with ARV prophylaxis and decreasing MTCT rates, against a background of increasing annual enrolments. The absolute numbers of IDU women enrolling in the study actually increased in 2007 compared to 2006 (data not shown), but we report a declining proportion of women with IDU history over time, reflecting trends towards increasing heterosexual transmission in the Ukraine [3,15]. MTCT rates more than halved between 2001 and 2006, with around one in fourteen women transmitting infection to their infants in 2006.

Prompt identification of HIV-infected pregnant women is essential for a successful PMTCT programme [12]. Around one in four women here were aware of their HIV diagnosis before conception, and nearly three-quarters had been diagnosed by the start of their third trimester. Women with IDU histories or IDU sexual partners and those with HIV symptoms were more likely to have a pre-pregnancy HIV diagnosis than other women which probably indicates their increased likelihood of accessing voluntary counseling and testing (VCT) outside pregnancy compared with other women. The increased likelihood of pre-pregnancy diagnosis in 2006/07 versus 2000/01 suggests improvements in VCT coverage over time; a national protocol for VCT was not approved by the Ministry of Health until December 2005, which is consistent with this time trend. Access to harm reduction programmes for IDUs increased nationwide within the framework of a Global Fund supported programme, particularly during 2005–2007, which is also likely to have contributed to increasing coverage of VCT for IDUs, including female IDUS.

Among women becoming pregnant with unknown HIV status, IDUs were more than 3.5 times more likely to be diagnosed through intrapartum rapid testing than women with a non-IDU-related mode of acquisition, indicating reduced access to antenatal care and thus the application of less effective PMTCT interventions. It is well recognised that this marginalised group of women frequently experience problems accessing health and social services [22,23]. In a study in St Petersburg, Russian Federation among women presenting in labour with unknown HIV status, largely without antenatal care, two-thirds were IDUs [24]; HIV seroprevalence was 6.5% among women without antenatal testing, substantially higher than the 1–2% among women accessing antenatal care [25,26].

Our results indicate the greater effectiveness of antenatal ZDV compared with sdNVP in PMTCT, consistent with previous findings [12]. As the few women receiving antenatal HAART had clinical and/or immunological indications for HIV treatment, which are associated with increased MTCT risk [9], one might potentially expect a greater reduction in transmission risk than the 84% seen here associated with HAART use if HAART were implemented as prophylaxis for all women [13,15], which is planned for Ukraine's next PMTCT programme, from 2009. Around 18% of women delivering in 2007 received sdNVP alone or with ZDV. Although current WHO guidelines [12] recommend that where possible a 3TC and ZDV "tail" should be provided postnatally for mothers receiving sdNVP, to reduce the likelihood of NVP resistance developing [16], this was not included within the Ukraine PMTCT programme. It remains unclear to what extent NVP resistance could compromise success of subsequent treatment of mother and child with NNRTI-containing regimens [27-30].

Elective CS was an effective PMTCT intervention in this setting, associated with a near-halving of risk, consistent with earlier results in Western Europe [14,31-33]. However, we documented considerable variation in the application of this PMTCT intervention across our study sites. Ukraine is a setting where formula feeding is acceptable, feasible, affordable, sustainable and safe and this is recommended for all HIV-positive women within the national policy. Although free breast milk substitutes are theoretically available, there are historic reports of limited access for some women [18,34]. Only around 1% of infants were breastfed, mostly for short durations. Breastfeeding was associated with a three-fold increased MTCT risk, although we were unable to determine the timing of transmission among HIV-positive breastfed infants. These findings are a pertinent reminder that appropriate feeding counselling and support for HIV-infected women [35] are needed even in settings where avoidance of breastfeeding is not generally perceived as problematic.

The halving of the MTCT rate since 2001 documented here marks the success of the national PMTCT programme, although key challenges remain which must be addressed if the country is to achieve the Dublin Declaration target of "virtual elimination" of HIV transmission to infants by 2010 [36]. Women least likely to receive ARV prophylaxis included IDUs and those diagnosed intrapartum, which mirrors findings from elsewhere in Europe [37-39]. Efforts are needed to address barriers that these women may face in accessing services, particularly as many may have concurrent infections, including hepatitis C [40] and sexually transmitted infections [41]. Linkages between harm reduction, oral substitution therapy and PMTCT services need to be established, with development of services targeted at hard-to-reach IDU pregnant women. It will also be important to improve quality of PMTCT interventions, including better access to CD4 count monitoring and HAART and improved coverage of early infant diagnosis. Primary prevention of HIV acquisition in women is the most effective approach for preventing infections in infants [11,23], but services in Ukraine remain under-developed, both those directed to at risk populations, such as IDUs, and for the general population. Nearly half of the women here reported no specific risk factors for HIV and most likely acquired infection heterosexually. As Ukraine has an HIV prevalence exceeding 1.5%, increasing heterosexual transmission and a young HIV-positive population (three-quarters aged 30 or less) [8,42], it is essential that concerted effort is directed towards primary prevention, as recommended by the recent UNAIDS-coordinated External Evaluation of the National AIDS Response [38].

Our study was limited by the observational nature of the data and although we adjusted for confounding factors in our multivariable analyses, there is potential for unmeasured confounding. Other limitations included the paucity of maternal CD4 counts and lack of HIV RNA measurements, which were not widely available within routine clinical practice. In a setting with limited access to early diagnosis for HIV-exposed infants, scope for loss to follow-up before infection status can be determined is considerable. Here, 15% of infants had indeterminate infection status despite being aged >18 months; applying the relevant transmission risks for the five strata of ARV prophylaxis applicable to these 535 children, we estimate that an estimated 65 (12.1%) would be infected. Adding this figure to the 1690 children with known infection status gives an overall MTCT estimate of 11.6% (c.f. 11.4% reported). Regarding the generalizability of our results, of the estimated 2731 HIV-infected women who delivered nationally in 2007 [38], nearly a third were enrolled in the ECS, suggesting that our cohort is representative of the HIV-infected pregnant population in Ukraine. Of note, as the ECS only includes women who deliver, characteristics may differ from women terminating their pregnancies.

## Conclusion

Data from our study not only provide valuable information for the evaluation and strengthening of Ukraine's PMTCT programme, but also for the ongoing development of strategies for prevention of HIV infection in infants elsewhere in Eastern Europe and Central Asia. The experience in Ukraine highlights that it is possible for a lower income country to make a substantial impact on MTCT (in this case, having of the MTCT rate in only five years) and underscores the appropriateness of the public health approach to prevention of HIV infections in infants recommended by WHO [10] and adopted in Ukraine with obvious success. Future research should focus on hard-to-reach populations such as IDU, in addition to the urgent issues of primary prevention and prevention of unintended pregnancies among HIV-positive women. In particular, scaling-up prevention of heterosexual transmission of HIV from and among IDUs will contribute to PMTCT in Ukraine.

## Competing interests

The authors declare that they have no competing interests.

## Authors' contributions

CT and RM contributed to study concept and CT, RM, IS and TP contributed to study design. RM, IS and TP were involved in the acquisition of data. CT drafted the manuscript and performed the statistical analyses. The Ukraine European Collaborative Study Group contributed to the design and/or data collection for this study. All authors critically revised the manuscript for important intellectual content and read and approved the final manuscript.

## Funding

The ECS is a coordination action of the European Commission (PENTA/ECS 018865). Claire Thorne is supported by a Wellcome Trust Research Career Development Fellowship. Some of this work was undertaken at GOSH/UCL Institute of Child Health which received a proportion of funding from the UK Department of Health's NIHR Biomedical Research Centres funding scheme. The Centre for Paediatric Epidemiology and Biostatistics also benefits from funding support from the Medical Research Council in its capacity as the MRC Centre of Epidemiology for Child Health.

## Pre-publication history

The pre-publication history for this paper can be accessed here:

http://www.biomedcentral.com/1471-2334/9/40/prepub
